# How the technologies behind self‐driving cars, social networks, ChatGPT, and DALL‐E2 are changing structural biology

**DOI:** 10.1002/bies.202400155

**Published:** 2024-10-15

**Authors:** Matthias Bochtler

**Affiliations:** ^1^ International institute of Molecular and Cell Biology in Warsaw Warsaw Poland; ^2^ Institute of Biochemistry and Biophysics Warsaw Poland

**Keywords:** DDPM, deep learning, inverse folding, LLM, neural net, protein compound interaction, protein design, structure prediction

## Abstract

The performance of deep Neural Networks (NNs) in the text (ChatGPT) and image (DALL‐E2) domains has attracted worldwide attention. Convolutional NNs (CNNs), Large Language Models (LLMs), Denoising Diffusion Probabilistic Models (DDPMs)/Noise Conditional Score Networks (NCSNs), and Graph NNs (GNNs) have impacted computer vision, language editing and translation, automated conversation, image generation, and social network management. Proteins can be viewed as texts written with the alphabet of amino acids, as images, or as graphs of interacting residues. Each of these perspectives suggests the use of tools from a different area of deep learning for protein structural biology. Here, I review how CNNs, LLMs, DDPMs/NCSNs, and GNNs have led to major advances in protein structure prediction, inverse folding, protein design, and small molecule design. This review is primarily intended as a deep learning primer for practicing experimental structural biologists. However, extensive references to the deep learning literature should also make it relevant to readers who have a background in machine learning, physics or statistics, and an interest in protein structural biology.

## Graphical abstract

Deep learning, a brain inspired field of computer science, revolutionizes structural biology. I summarize how convolutional neural networks (CNNs), large language models (LLMs), denoising diffusion probabilistic models (DDPMs)/Noise conditional score networks (NCSNs), and graph neural networks (GNNs) have impacted protein structure prediction, inverse folding, protein design, and small molecule design.

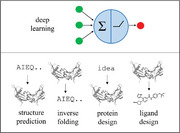

## INTRODUCTION

Deep NNs (NNs) are revolutionizing many areas of human endeavor.^[^ ^1^ ^]^ Self‐driving cars equipped with convolutional NN (CNN) vision ^[^ ^2^, ^3^ ^]^ and sophisticated decision‐making systems rival the capabilities of human drivers.^[^ ^4^ ^]^ Large Language Models (LLMs) have reached a level of sophistication ^[^ ^5^ ^]^ that makes it possible to greatly improve the quality of writing in the same language, to produce high‐quality translations from any language to any other language,^[^ ^6^ ^]^ and to perform tasks that require text understanding, such as text continuation (“next word prediction”), text sentiment analysis,^[^ ^7^ ^]^ or human‐readable software review and annotation.^[^ ^8^ ^]^ LLMs such GPT ^[^ ^9^, ^10^, ^11^ ^]^ have demonstrated reasoning power that is competitive with humans for many tasks,^[^ ^10^, ^11^ ^]^ including academic tasks such as passing the Wharton Business School exam.^[^ ^12^ ^]^ Combined with reinforcement learning, LLMs can even solve Mathematics Olympiads problems, and have recently won a silver medal in the annual competition.^[^ ^13^ ^]^ Graph neural networks (GNNs) power social media algorithms, and influence our experience with these media. Finally, denoising diffusion probabilistic models (DDPMs), such as DALL‐E2, and related noise conditional score networks (NCSNs) have demonstrated astonishing abilities to generate high‐quality, high‐resolution, language‐conditioned images in any desired style.^[^ ^14^, ^15^, ^16^, ^17^, ^18^ ^]^

**FIGURE 1:**
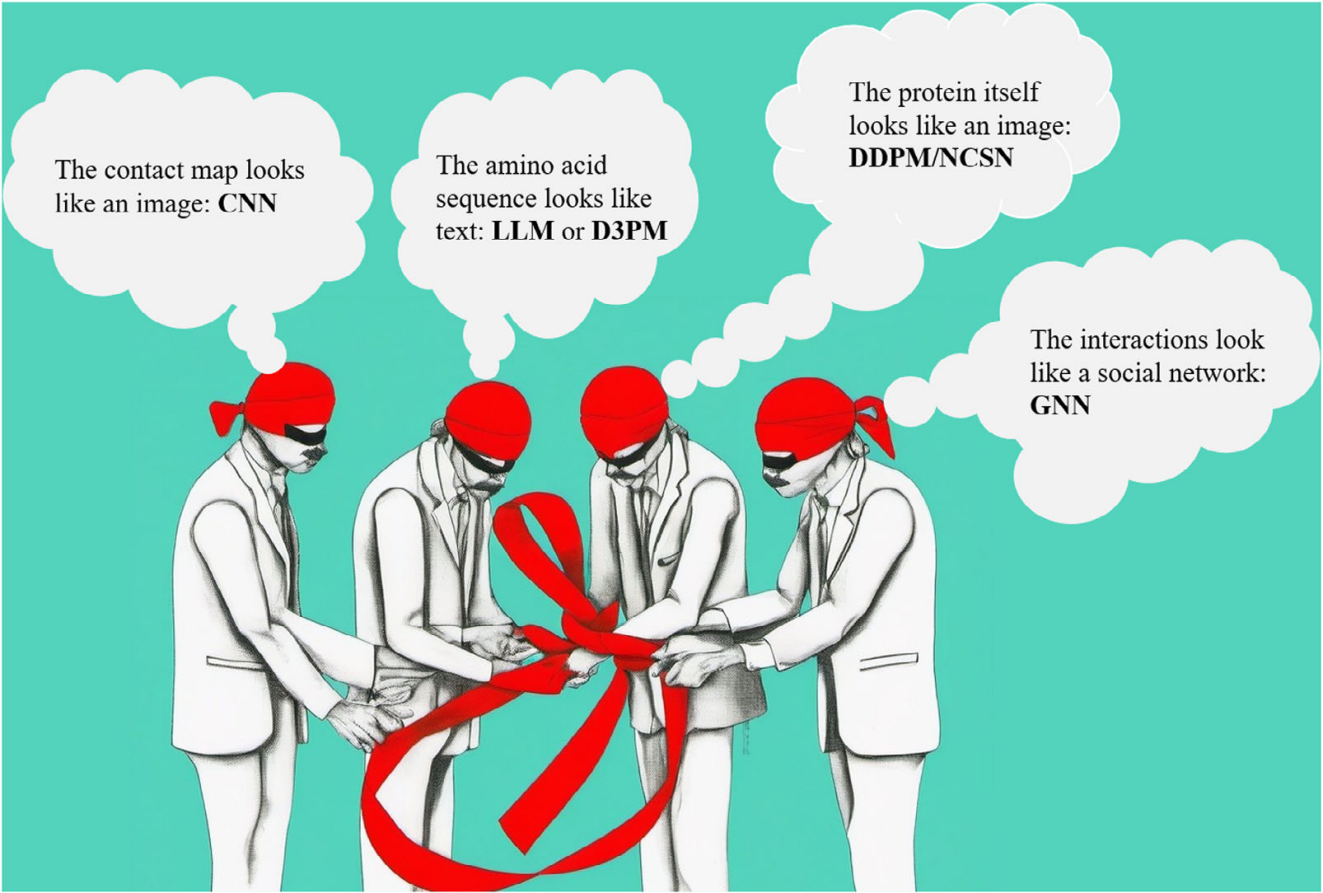
**Different perspectives on a protein**: The four blind men represent four different points of view of a protein that capture different aspects of its “true nature.” Each point of view connects protein structural biology with a different area of machine learning, ranging from CNNs to LLMs, D3PMs, DDPMs/NCSNs, and GNNs. The initial image was generated by a DDPM model based on the language prompt “four blindfolded men touching a protein model,” and then edited manually.

Not surprisingly, all these developments have also impacted structural biology. Protein contact maps,^[^ ^19^ ^]^ which contain information about protein structure, look like images, and thus call for the use of CNNs. The amino acid sequences of proteins can be regarded as “texts” in the “language” of proteins, suggesting the use of LLMs for their analysis. From a structural perspective, proteins with their short and long range interactions resemble social networks, suggesting that GNNs could be used to study them. Finally, the gradual emergence of molecular structure from a random and meaningless initial conformation during protein (re)folding is very reminiscent of the way in which DDPMs generate high‐resolution images from noise (Figure 1).

**FIGURE 2:**
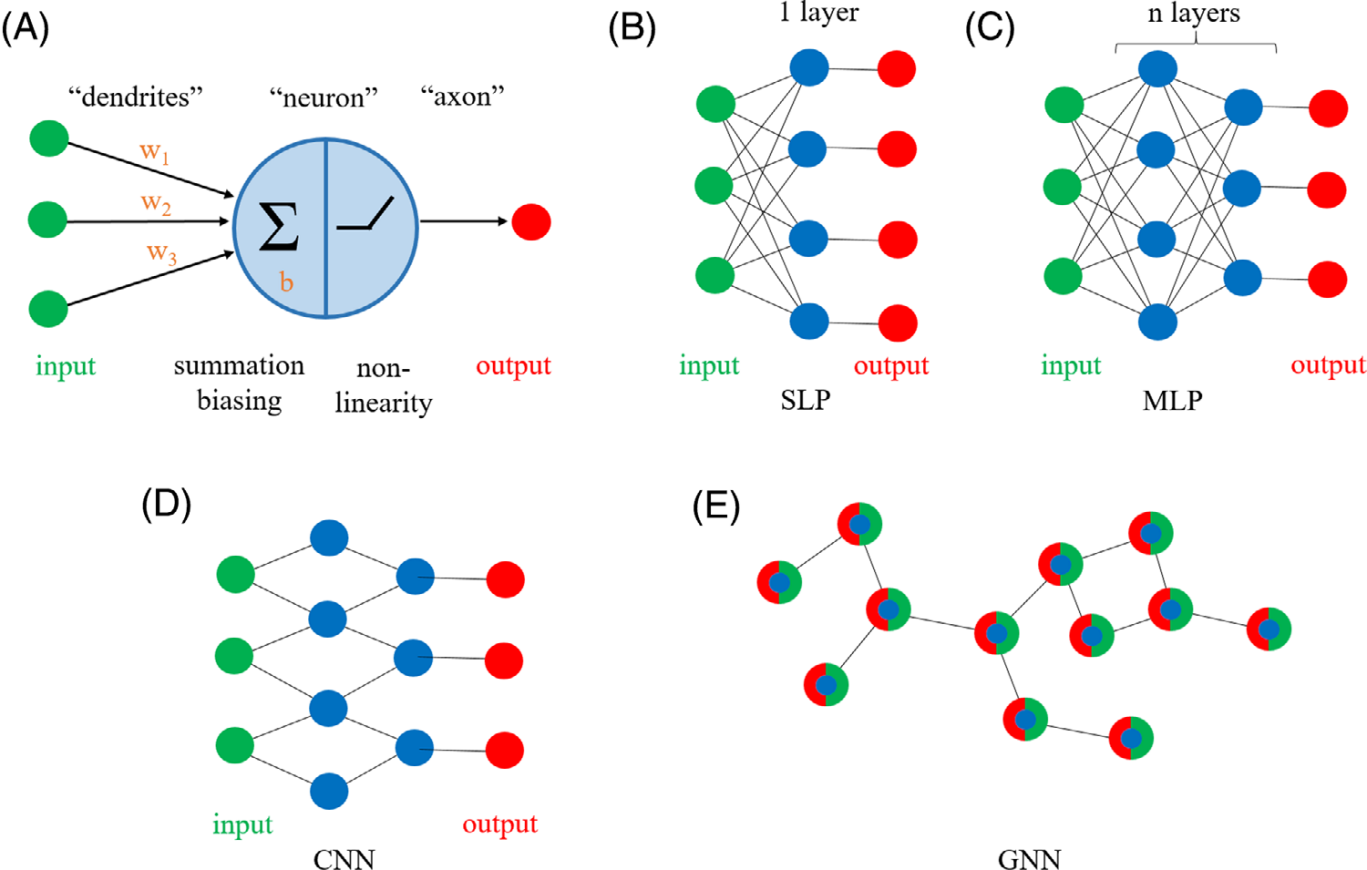
**NNs**: (A) The basic computational unit of NNs takes multiple inputs (green), multiplies them by learned weights (here: w1, w2, w3, brown), adds them (Σ), adds a learned bias (here: b, brown), and then applies a nonlinearity (here a RELU function ^[^ ^177^ ^]^, other choices are possible ^[^ ^178^ ^]^) to produce an output signal (red). The structure is designed to mimic the architecture of a neuron that receives information from multiple sources via its dendrites to generate an output that is sent down an axon. The learning of weights is reminiscent of the optimization of synaptic strengths in the brain to produce a desired output. (B) SLP: A set of neurons processes inputs independently to produce outputs. The neurons do not interact with each other and together form a layer. (C) MLP: multiple layers of neurons are stacked on top of each other, with outputs from one layer serving as inputs to the next layer. The number of layers and the number of neurons per layer are hyperparameters that are not learned. Since every neuron in a layer is connected to every neuron in the next layer, the network is “densely connected.” (D) CNN: A CNN network has only connections that aggregate local information (here from at most two adjacent inputs). The example is in 1 dimension. The most typical application is in 2 dimensions, for image analysis. (E) GNN: A graph convolutional network generalizes the concept of local information aggregation to irregular structures. The neighborhood is defined based on the edges connecting the nodes (vertices) of the graph. All layers of a GNN preserve the graph structure of the input. The learnable parameters influence how nodes update each other between layers (see Figure 5).

**FIGURE 3:**
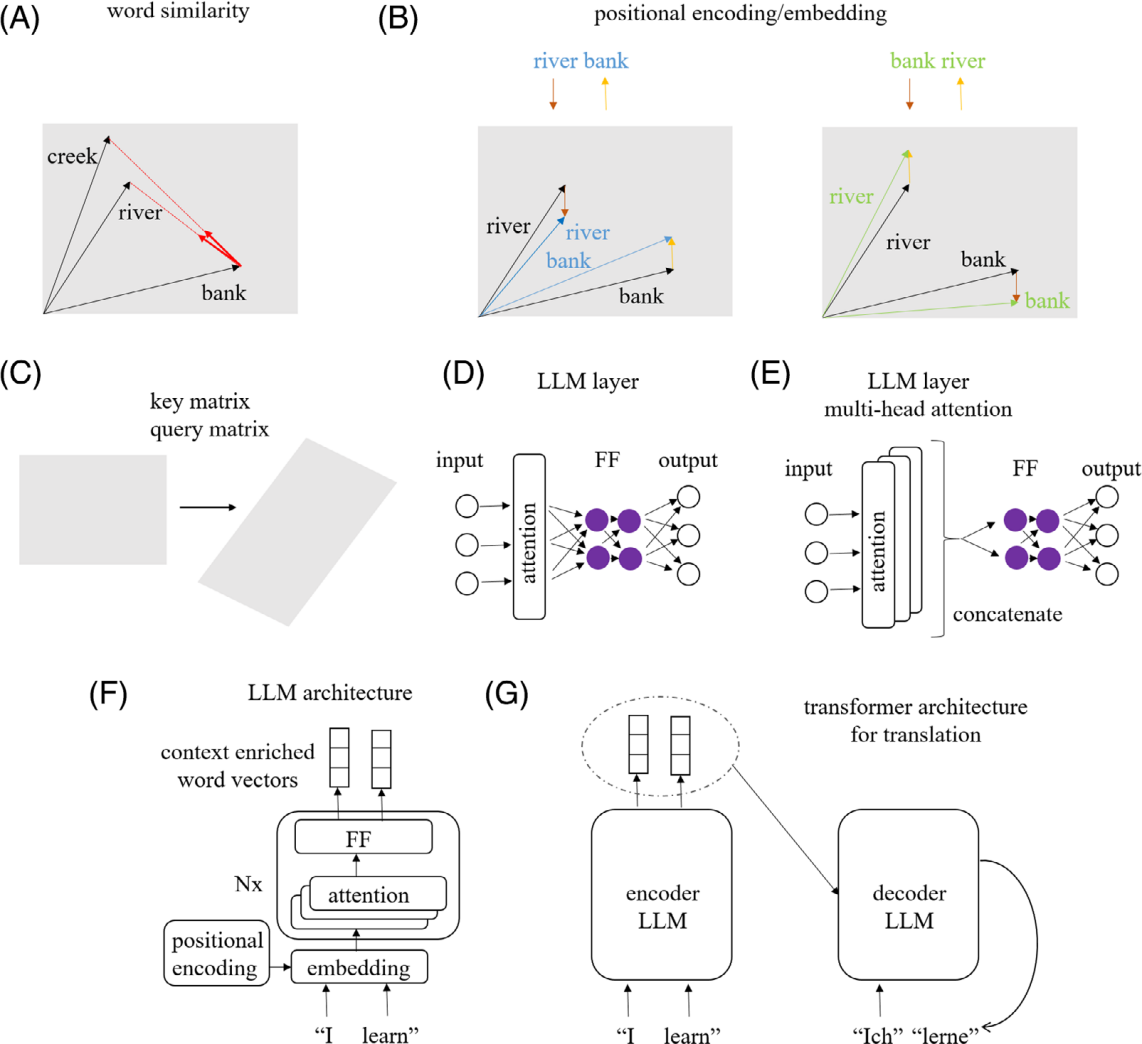
**Attention, LLMs and transformers**: (A) Words (or building blocks of words, so called tokens) are embedded by tools such as word2vec into a vector space.^[^ ^179^ ^]^ “Similar” words are represented by “similar” vectors. The attention mechanism lets words of a sentence (or any piece of text of fixed length) influence each other to generate context‐enriched word vectors (red arrows). (B) Positional information (brown and orange arrows) is used to modify the embedding vectors according to word position (i.e., to alter the black word vectors to the blue and green vectors). (C) Word vectors are transformed linearly by the (learnt) query and key matrices to optimize the expressivity of the vector space, thus generating query and key vectors. A similar transformation is applied to the value vectors that are used for the update procedure (not shown). (D) An attention layer followed by an MLP or feed‐forward (FF) layer (technical details such as normalization layers and skip connections are omitted). (E) In practice, multiple attention layers (“heads”) are used at the same time, and their outputs are concatenated before input to the MLP. (F) LLM architecture. “Nx” stands for N repeats of the unit (G) Transformer architecture: An autoregressive decoder LLM is conditioned on a “standard” encoder LLM.

**FIGURE 4:**
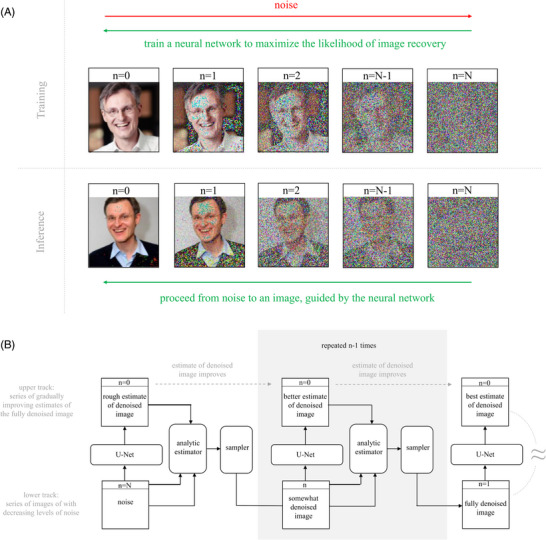
**DDPMs**: (A) Working principle: Training images are gradually noised, until the images are almost pure noise (red). The noising process can be understood as a form of data augmentation that supplements each training image with additional noisier versions. An NN is trained to maximize the log likelihood of the (augmented) training set of images ^[^ ^39^ ^]^, or a surrogate, the evidence lower bound (ELBO) of the image likelihood.^[^ ^14^, ^15^ ^]^ For inference, noise is gradually converted into an image (green). (B) Inference: Starting from a partially denoised image, an NN (typically a U‐net ^[^ ^28^ ^]^) is used to estimate the original image (or, equivalently, the noise in the input image).^[^ ^15^, ^38^ ^]^ When the input image is noisy, the estimation of the denoised image is imperfect. Nevertheless, the imperfectly denoised image can be input (together with the input image and the iteration step n) to an analytic an estimator to compute the expectation for an image that is slightly less noisy than the input image.^[^ ^39^ ^]^ A sampler then chooses a new, updated input image based on this expectation (grey box). The process is initiated from noise (left), and repeated multiple times. Conceptually, a DDPM generates two series of images, an upper track with increasingly better estimates of the fully denoised image, and a lower track containing images with less and less noise. As more iterations are performed, the top and bottom tracks converge to the same image (as indicated by the ≈ symbol). Note that inference runs from right to left in panel (A) for consistency with other presentations of the DDPM working principle, and from left to right in panel (B), for consistency with how autoregressive networks are usually depicted.

**FIGURE 5:**
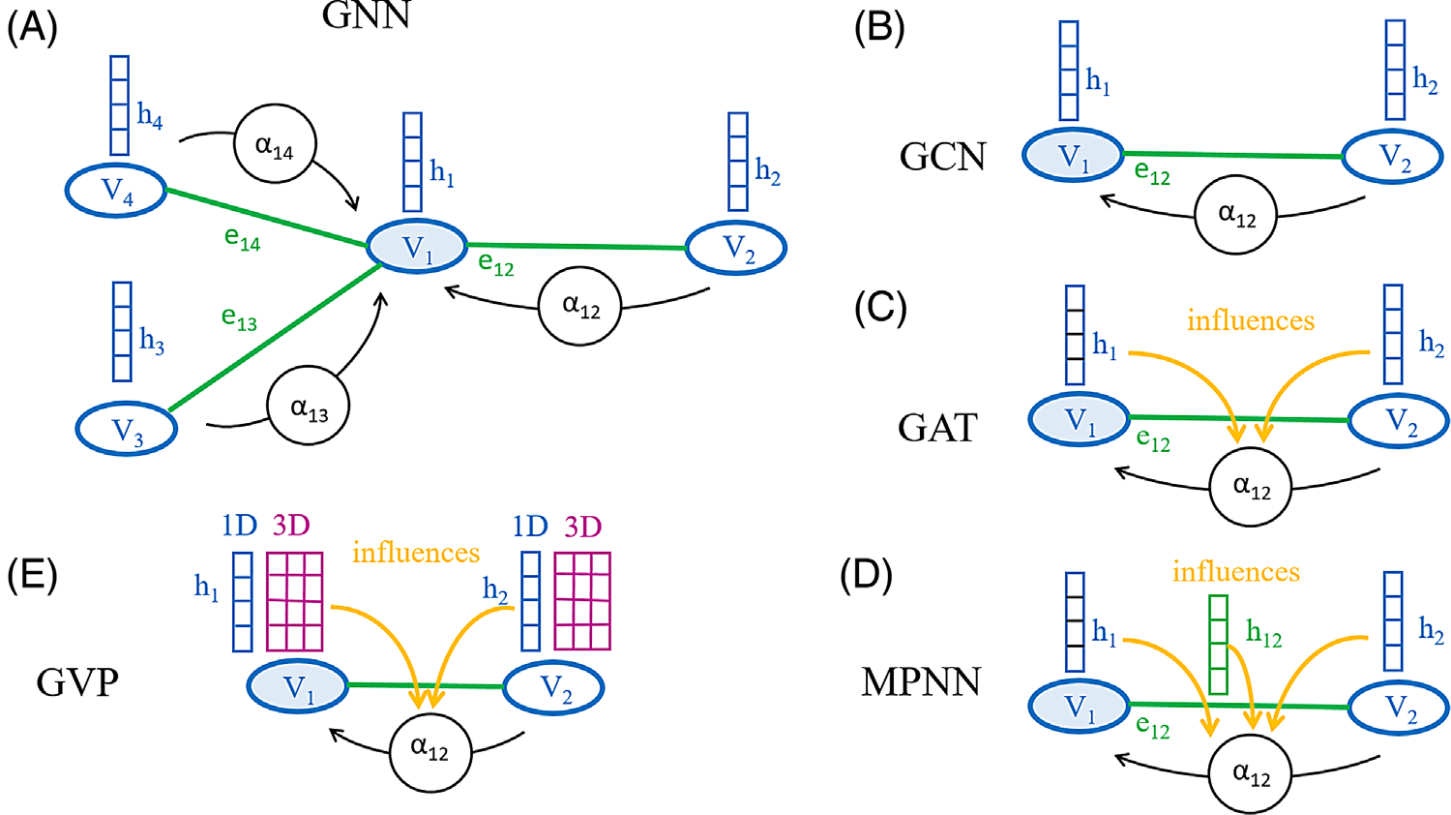
**GNNs**: GNNs operate on graphs that consist of nodes (also called vertices) (V_i_) and edges (e_ij_). Nodes V_i_ are associated with hidden state vectors h_i_ that are updated based on messages from adjacent nodes by every layer of the network. (A) Node update: the hidden state of a node, here h1 of V_1_ (light blue) is updated based on the hidden states of nodes (here V_2_, V_3_, V_4_) that share an edge with the node of interest. Updates require a weighting of the neighboring hidden states by edge specific coefficients α, aggregation (elementwise sum or maximum), a projection, and a non‐linearity. The GNNs differ in how the aggregation weights are determined. (B) In GCNs, the weights α do not depend on the hidden states. (C) In GATs, the weights α are dependent on the hidden states (orange arrows). (D) MPNNs extend GATs by associating hidden states also with edges. (E) GVPs are like GATs, but hidden states consist of separate arrays for 3D scalars (as in GATs, blue) and 3D‐vectors (purple) that are updated by an update rule that respects equivariance.

This review begins with a brief overview of deep learning methods that have had a major impact on structural biology (Table 1). The neuron as the basic compute unit is explained (Figure 2A), and basic neural network architectures such as CNNs (Figure 2D), LLMs (Figure 3), DDPMs/NCSNs (Figure 4), and GNNs (Figure 2E and Figure 5) are introduced. The reminder of the manuscript builds on this machine learning background to describe how these technologies have impacted protein structure prediction (Figure 7), inverse folding (Figure 8A‐B), protein design (Figure 8C), and small molecule ligand design.

**TABLE 1:** Machine learning glossary.

NN	Neural network
SLP	Single‐layer perceptron
MLP	Multi‐layer perceptron
CNN	Convolutional NN
LLM	Large language model
GNN	Graph NN
GCN	Graph convolutional network
GAT	Graph attention network
GVP	Geometric vector perceptron
MPNN	Message passing NN
DDPM	Denoising diffusion probabilistic model
DDIM	Denoising diffusion implicit model
D3PM	Discrete DDPM
LDM	Latent diffusion model
NCSN	Noise conditional score network
SD	Stable diffusion
GAN	Generative adversarial network

**Box 1 How attention changes the vector embedding of words (or tokens) in transformer LLMs**

LLMs work with a representation of words (or tokens) as vectors in a high‐dimensional vector space. The vectors are calculated by an embedding algorithm such as word2vec which assures that “similar” words are placed in similar regions of the vector space.^[^ ^169^ ^]^ “Similarity” between word vectors (and thus words) is quantified by one of three related measures. Cosine similarity is defined as the cosine of the angle between the vectors (with maximal value 1 when vectors are collinear). Scalar (dot) product similarity is cosine similarity multiplied by the length of the vectors, which is equal to the scalar (dot) product between the vectors. Scaled scalar (dot) product similarity, the most widely used similarity measure, scales scalar (dot) product similarity by the root of the number of vector dimensions. The attention mechanism ^[^ ^6^ ^]^ contextualizes words, in the space of word vectors. Conceptually, the similarities of any given word to all other words are calculated, and the set of similarity scores is then subjected to exponentiation and normalization (together a softmax function ^[^ ^27^ ^]^) to calculate update weights. The new word is the weighted sum of all word vectors. The procedure ensures that word vectors do not grow on average, and that contributions of all word vectors are always positive (Figure 3A). In practice, several modifications to this simple idea are used in the attention blocks of LLM transformers. First, word order matters, yet the algorithm described so far is insensitive to word order. Therefore, positional encodings (sometimes also termed positional embeddings) can be used to modify word vectors.^[^ ^6^, ^170^ ^]^ Their exact form can vary: They can encode absolute or relative word position, and be fixed or learned.^[^ ^171^ ^]^ In all cases, they are used to modify word vectors according to word position (Figure 3B). Second, learned linear transformations are applied to word vectors to maximize the expressiveness of word comparisons and the effectiveness of the update (Figure 3C). Moreover, before summation to calculate new word vectors, the word vectors are subjected to another linear transformation to calculate the so‐called value vectors (not shown). Outputs from attention layers are fed to a feed forward (FF) module (essentially an MLP without the last non‐linearity) (Figure 3D). Multiple attention layers (called “heads”) can operate on the same input, to focus on different aspects of words during the update procedure (Figure 3E). An LLM is essentially a stack of attention layers operating on word vectors with positional encoding (Figure 3F). For translation, the transformer architecture with “standard” encoder LLM and auto‐regressive decoder LLM is used (Figure 3G).

### A deep learning primer for structural biology experimentalists

The basic computational unit of NNs is called the “neuron,” based on its similarity to neurons in the brain. A computational neuron gathers multiple inputs (from “dendrites”), weights and aggregates them, adds a bias, and then applies a nonlinearity (the “firing threshold”) to produce an output (into an “axon”).^[^ ^20^, ^21^ ^]^ Like synaptic strengths, weights and biases are tunable (“learnable”) parameters. Their values are chosen to minimize discrepancies between actual and desired outputs (Figure 2A). A set of neurons that independently process the same inputs is termed a Single‐Layer Perceptron (SLP) (Figure 2B). The limited capabilities of SLPs ^[^ ^22^ ^]^ increase drastically when they are stacked to create Multi‐Layer Perceptrons (MLPs) (Figure 2C). The learnable parameters of MLPs can be tuned by the backpropagation algorithm,^[^ ^23^ ^]^ which generalizes the chain rule of calculus, to make the network generate desirable outputs.

MLPs are fully connected networks, that is, every neuron in one layer is connected to every neuron in the next layer. This architecture is unnecessarily expensive computationally when inputs have strong local correlations. In such cases, it is advantageous to let the network aggregate information locally. By stacking multiple locally operating layers, long‐range interactions can eventually also be captured. As the local aggregation operation is a convolution, such networks are known as CNNs ^[^ ^24^, ^25^ ^]^ (Figure 2D). In case of CNNs, the inputs are regularly spaced, on a line in the 1D‐case, or in a rectangle in the 2D‐case. GNNs generalize the notion of neighborhood to irregularly spaced inputs ^[^ ^26^ ^]^ (Figure 2E).

### CNNs for computer vision

CNNs are at the heart of computer vision.^[^ ^24^, ^25^ ^]^ Convolutions aggregate information locally, based on learned rules (“kernels”). Typically, more than one learned kernel is used at any stage, which increases feature dimensions (channels). At the same time, the aggregation typically reduces image size. Eventually, a multi‐channel 1 × 1 “image” is created that can be processed by a softmax function ^[^ ^27^ ^]^ to pseudo‐probabilities for class labels. Variants of CNNs can also be used for image segmentation instead of image classification. As the architecture of such networks is often described by a “U”‐shaped diagram, such nets are known as U‐Nets.^[^ ^28^ ^]^ They have many applications in computer vision, beyond their original use in medical image analysis, including in image generation. After decades of dominance in image analysis, the CNNs have recently faced serious competition from vison transformers (ViT), which adapt LLM technology to images.^[^ ^29^ ^]^

### LLMs with attention for language understanding and translation

LLMs have been developed for text‐related tasks, such as text understanding, summarization, or translation. They are based on the concept of “attention”.^[^ ^6^ ^]^ The key idea of attention is that context can be used to refine the meaning of words (or syllables) and to build richer representations of words in an abstract space by iteratively refining the representation based on neighbors (Figure 3 and Box 1). For example, after a single round of refinement, the representation of the word “bank” in the sentences “the bank was closed” and “the bank of the river was covered with mud” will be different. Multiple iterations of word refinement are usually helpful. Therefore, LLMs such as BERT use stacked attention layers.^[^ ^30^ ^]^ LLMs learn on unlabeled data (raw non‐annotated text), with tasks such as masked token or next word prediction. Modern versions of LLMs often exploit similarities between languages and are trained on multiple languages simultaneously.^[^ ^31^, ^32^, ^33^ ^]^ The content enriched word vectors from the top layers of an LLM can be used as input for NNs that carry out more specialized tasks such as sentiment analysis or spam detection, without or with only minimal fine‐tuning of the LLM trainable weights. For the special task of language translation, transformers are used.^[^ ^6^ ^]^ They consist of two LLMs that are termed the encoder and the decoder. The encoder is run once, and generates an array of word vectors that represent the input text. The decoder is conditioned on this encoder output, and runs autoregressively to determine the translated text word by word.^[^ ^6^ ^]^

### DDPMs and NCSNs for unconditional and conditional image generation

DDPMs ^[^ ^14^, ^15^, ^34^ ^]^ excel at image generation. They were popularized by OpenAI's DALL‐E2,^[^ ^17^ ^]^ and later by Stability AI's Stable Diffusion (SD) text‐to‐image systems.^[^ ^35^, ^36^ ^]^ DDPMs learn a distribution of the images from samples, at various levels of “smoothing” by added noise.^[^ ^15^, ^37^, ^38^ ^]^ The noising process may be viewed as a random walk in “image space” and understood as a form of “data augmentation”.^[^ ^39^ ^]^ The reverse of the noising process, denoising, generates new images (Figure 4A). For each level of noise, a U‐Net ^[^ ^28^ ^]^ or ViT ^[^ ^40^ ^]^ is used to predict the denoised image, or equivalently the noise in the input image. This prediction is imperfect, but it is good enough to let an analytic estimator determine the expectation for a slightly less noisy image, taking into account also the current input image and denoising stage. Based on this expectation, a sampler then selects the next input image for another iteration. The auto‐regressive image computation is started from noise, and eventually arrives at a fully denoised image after many iterations (Figure 4B). As inference with the original DDPM architecture requires many successive denoising steps, it is computationally expensive. Consistency models allow to skip denoising steps and to choose between image quality and computational cost.^[^ ^41^ ^]^ Denoising diffusion implicit models (DDIMs) improve on the noising process to make denoising more efficient.^[^ ^42^ ^]^ Diffusion can also be carried out for compressed latent space images.^[^ ^36^ ^]^ The corresponding models are known as Latent Diffusion Models (LDMs) and power SD.^[^ ^35^, ^36^ ^]^

DDPMs were originally developed for images, and are therefore best suited for continuous (or ordinal) data. However, key concepts from DDPMs can be modified to fit categorical data. The resulting networks are called discrete DDPMs, or D3PMs.^[^ ^43^, ^44^, ^45^ ^]^ Unlike the DDPMs, the D3PMs are suitable for tasks that are traditionally considered the domain of LLMs. Score entropy discrete diffusion (SEDD) models, which can be regarded as improved D3PMs, perform comparably to ChatGPT2 in the language domain, for similarly sized networks.^[^ ^46^ ^]^

NCSNs are conceptually similar to DDPMs, but without noise level discretization.^[^ ^18^ ^]^ Just like DDPMs, they are powered by U‐Nets ^[^ ^28^ ^]^ or variants thereof.^[^ ^18^, ^47^ ^]^ However, the prediction target for these NNs is different. While DDPM NNs predict the original image, or the noise in the current version of the image, the NNs in NCSNs predict a score (the Stein score) to guide the reverse diffusion process,^[^ ^18^, ^48^ ^]^ or its deterministic equivalent,^[^ ^16^ ^]^ from noise to images.

Without the ability to control the image generation process, DDPMs would be of limited use. Fortunately, there are many effective ways to bias the computed images by text prompts. One way is to embed the text prompts and images in the same latent space.^[^ ^17^ ^]^ Alternatively, text prompts can bias computed images by cross‐attention,^[^ ^36^ ^]^ or by biasing diffusion steps based on classifier gradients.^[^ ^37^ ^]^ Finally, image/noise predictors can be trained to use or to disregard text prompts, to select diffusion steps that favor text‐prompt consistent over generic directions in image space.^[^ ^49^ ^]^ Text prompt driven image generation may be viewed as a form of text‐to‐image “translation”.

### GNNs for the description of social networks (and other) interactions

GNNs are well suited for the description of urban traffic patterns, small molecules, or proteins with both long‐range and short‐range interactions. Relations between neurons (nodes, vertices) are defined by the presence or absence of graph edges. GNNs pass hidden state information between nodes by messages, that are aggregated (sum or maximum) (Figure 5A), projected according to learned parameters, and subjected to a non‐linearity. Depending on the weighting scheme of hidden states, graph convolutional networks (GCNs) (Figure 5B),^[^ ^26^ ^]^ graph attention networks (GATs) (Figure 5C),^[^ ^50^ ^]^ and message passing NNs (MPNNs) (Figure 5D) ^[^ ^51^ ^]^ can be distinguished. The interactions of hidden states in GATs and MPNNs are very similar to the interactions of word vectors in LLMs. Therefore, GATs and MPNNs can be considered as GNN generalizations of LLMs.^[^ ^52^ ^]^ In GCNs, GATs, and MPNNs, the hidden states are arrays of scalar values that do not change under rotations and translations. Geometric Vector Perceptrons (GVPs) have hidden states consisting of arrays of scalars and 3D‐vectors. Their update rule assures that 3D‐rotations can be applied with the same effect before and after the GNN operation. GNNs with this property are called equivariant networks (Figure 5E).^[^ ^53^ ^]^ So‐called “E(n) equivariant networks” combine elements of MPNNs and GVPs. They have hidden states associated with edges like MPNNs, and hidden state arrays of scalars and 3D‐vectors like GVPs (not shown).^[^ ^54^ ^]^ Allsubtypes of GNNs are permutation invariant: the results of GNN computations do not depend on the order in which the nodes are labeled. Like LLMs, GATs, MPPNs, GVPs, and E(n) networks can be run as encoders, or auto‐regressively as decoders, or as graph transformers with coupled graph encoders and decoders for “translation”‐like tasks.

### Protein structure prediction

Protein structure prediction has benefited dramatically from developments in the field of artificial intelligence. Protein models that are nearly as reliable as experimental structures can now be generated for almost any sequence of interest.^[^ ^55^ ^]^ Modeling of protein complexes is informative about structural aspects of protein‐protein interactions,^[^ ^56^ ^]^ and can even be used to screen in silico (albeit with a high false positive rate) for possible protein binders.^[^ ^57^ ^]^ For the detection of remote homology, comparisons of predicted structures are superior to sequence comparisons. On this basis, it has been argued that the traditional protein BLAST ^[^ ^58^ ^]^ may soon be replaced by structure‐aware search tools for remote homology detection.^[^ ^59^ ^]^

### Before the revolution: Template based modeling

Traditional protein structure modeling has mostly been based on the principle that phylogenetically related, similar sequences indicate a common, similar structure. This was the basis of widely used modeling tools such as I‐Tasser,^[^ ^60^ ^]^ X‐Raptor, ^[^ ^61^ ^]^ or Swiss‐model.^[^ ^62^ ^]^ In difficult cases, the best templates for fragments could be combined, as demonstrated by the success of Frankenstein's monster.^[^ ^63^ ^]^ The accuracy of traditional template‐based modeling depends strongly on the phylogenetic distance between template and target structure. For difficult cases, in the twilight zone of protein similarity,^[^ ^64^ ^]^ successful modeling requires the ability to reliably detect deep evolutionary relationships and to generate accurate alignments with minimal register error. Because of these requirements, template‐based modeling has always been, and still is, heavily dependent on evolutionary information. After dominating structure prediction for decades, template based modeling is gradually losing relevance.^[^ ^55^ ^]^ According to current benchmarks, it nowadays only makes sense when very good templates are available.^[^ ^65^ ^]^

### Contact maps from evolutionary information: Potts Models and AlphaFold1

**FIGURE 6:**
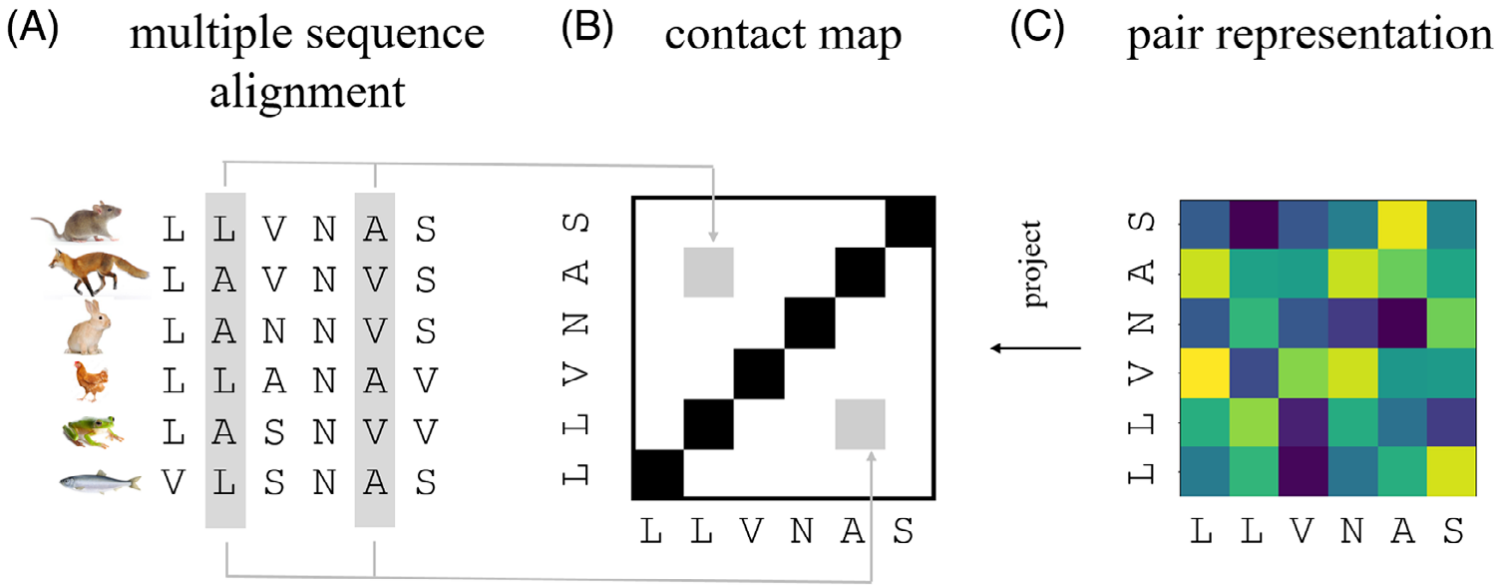
**Key ingredients for protein structure prediction**: (A) The multiple sequence alignment (MSA) contains evolutionary information about protein structure, in the form of covariation between homologous sequences from different species. In the example, the positions in grey co‐vary. An L in position 2 is always paired with an A in position 5, and an A in position 2 is always paired with a V in position 5. (B) The contact map shows predicted proximity, based on the co‐variation of residues in the MSA. Every residue is close to itself, hence the strong signal on the diagonal. (C) The pair representation can be understood as a generation of the contact map. Instead of a single scalar indicating proximity, the map contains a vector in a feature space for every pair of residues. This information is richer than the simple proximity information in the contact map. From the pair representation, a contact map can be projected out. Note that the pair representation is not necessarily symmetric with respect to the diagonal, unlike the contact map.

Multiple sequence alignments (MSAs) contain evolutionary information about structure (Figure 6A). The reason for this link between evolutionary and structural data is geometrical. The inside regions of proteins are closely packed.^[^ ^66^ ^]^ Therefore changes to one amino acid typically require compensatory changes to other amino acids in the vicinity. Thus, the co‐variation in the MSA, quantifiable by mutual information, can be used as a surrogate measure for residue proximity.^[^ ^67^, ^68^, ^69^ ^]^ The distance information is typically expressed as an NxN matrix (where is the number of residues in the protein) known as the contact map ^[^ ^19^, ^70^ ^]^ (Figure 6B). However, there is a catch: deducing contacts from co‐variation in sequence alignments directly is problematic. Suppose that position “A” in the alignment co‐varies with position “B” in the alignment due to proximity of residues “A” and “B,” and that position “B” in the alignment co‐varies with position “C”, also due to proximity. Mathematically, it is then inevitable that positions “A” and “C” co‐vary as well, without residues “A” and “C” necessarily being close to each other in space. Hence, to go from co‐variation to proximity, the contact map needs to be “refined,” to keep only direct interactions, and to weed out indirect ones. Traditionally, this has been done using Potts models,^[^ ^71^, ^72^ ^]^ which are generalizations of the Ising models.^[^ ^73^ ^]^ In a sense, AlphaFold1 can be viewed as a “glorified Potts model” that uses convolutional layers as in CNNs to discriminate better between direct and indirect co‐variation than a Potts model could.^[^ ^74^, ^75^ ^]^ The distance information from the refined contact map is then converted into a pseudopotential, which is further augmented by penalties for deviations from expected secondary structure and for “physics” violations.^[^ ^76^ ^]^ This composite potential is then used to guide structure refinement in torsion space. Conceptually, the path from distances to structure is similar to structure calculation from experimentally determined NMR distance and conformation restraints.^[^ ^77^ ^]^

### Combined reasoning over MSAs and pair representations: AlphaFold2, OpenFold, FastFold, RosettaFold

**FIGURE 7:**
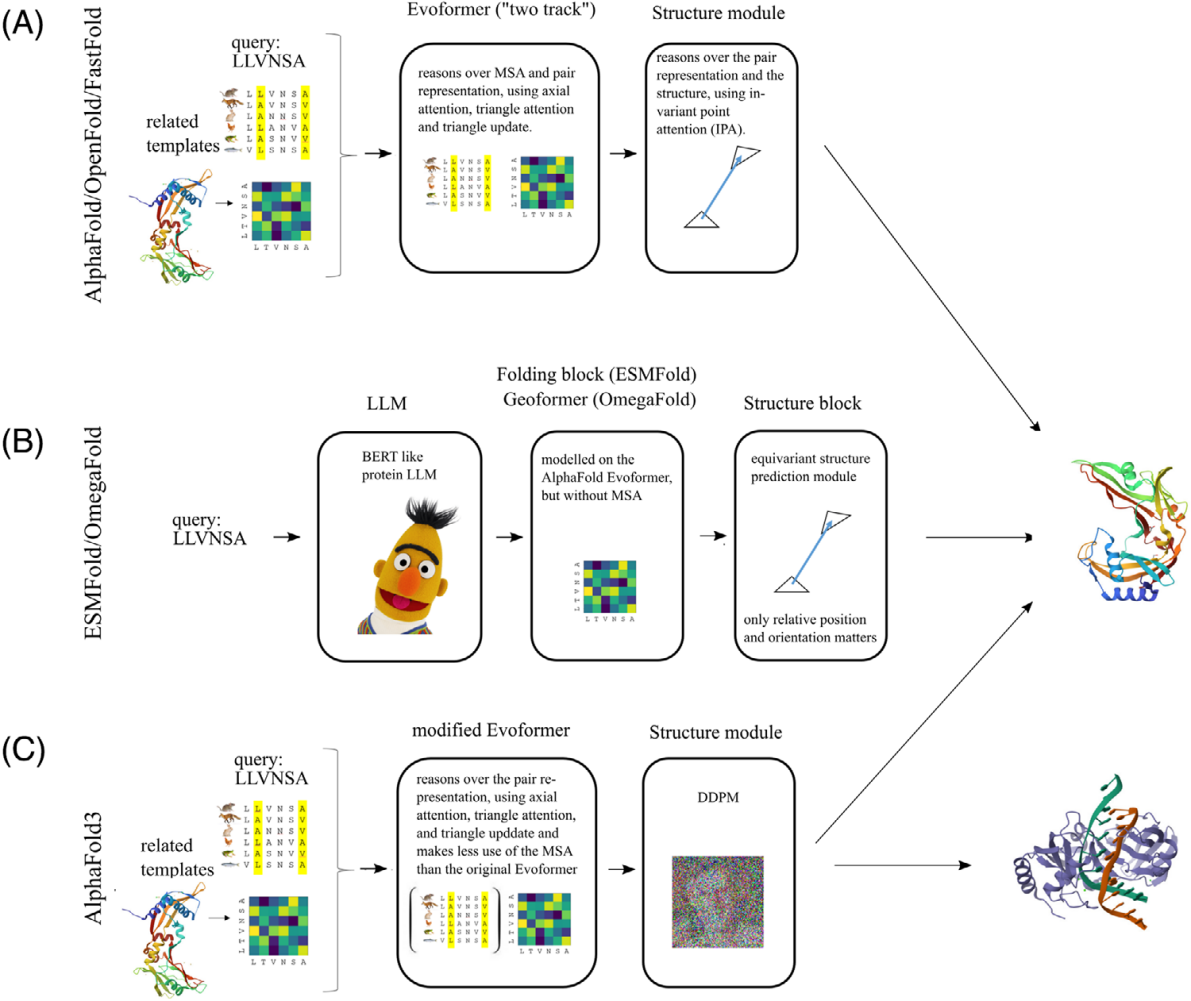
**Protein structure prediction tools**: (A) AlphaFold2/OpenFold/FastFold, (B) ESMFold/OmegaFold and (C) Alphafold3. With the exception of Alphafold3, the tools are only suitable for protein structure prediction. Alphafold3 is so far unique in also predicting also nucleic acids (both RNA and DNA), and small molecules, alone and in complex with proteins.

AlphaFold2 represents a radical departure from previously used protein structure prediction pipelines.^[^ ^55^ ^]^ Although it is an end‐to‐end differentiable system,^[^ ^78^ ^]^ AlphaFold2 is best described as a NN with two main blocks, the Evoformer and the Structure module (Figure 7A).

The Evoformer introduces several novel ideas. First, it generalizes the concept of a contact map to the “pairwise representation”, which contains a feature vector for each pair of amino acids (Figure 6C). Second, the MSA is no longer discarded after it has been used to compute a contact map or pair representation. Instead, it is retained and continuously refined as the model reasons jointly over the MSA and pair representation. The reasoning is attention‐based,^[^ ^6^ ^]^ with some modifications. As both the MSA and the pair representation are 2D, axial attention (i.e., row‐ and column‐wise attention) ^[^ ^79^ ^]^ is used instead of the standard attention to limit computational cost. More importantly, attention over the pair representation is modified to triangle self‐attention. Intuitively, this triangle self‐attention enforces the Euclidean triangle inequality that constrains possible arrangements of amino acids with respect to each other in space. As triangle self‐attention is computationally expensive, triangular update, a computationally cheaper, but similarly effective surrogate for triangle attention, is also used.^[^ ^55^ ^]^

The Structure module converts the abstract information in the pair representation into concrete coordinates. In a radical departure not only from Alphafold1, but from essentially all prior approaches, the amino acids of a polypeptide chain are treated as a residue gas of unconnected atoms. The model is aware of their linear arrangement, via the residue index. The model needs to learn to respect the linear arrangement, it is not forced to do so, except at the very end. Attention, already important for the Evoformer, is a key concept also for the Structure module.^[^ ^6^ ^]^ Here, it is also implemented in a spatially “invariant” flavor (called Invariant Point Attention, or IPA) that is not affected by translations and rotations. Protein structure is predicted iteratively in several steps, starting from an initial “black hole initialization.”

The whole structure prediction pipeline with Evoformer and Structure module can be looped so that predictions from the model become templates that can be used to initialize the pair representation for another iteration. The end result is very high quality predictions.^[^ ^55^ ^]^ By now, over 200 million structures have been pre‐calculated using Alphafold2,^[^ ^80^ ^]^ compared with only ∼0.22 million experimental structures in PDB ^[^ ^81^ ^]^ at the time of submission of this paper.

Since the success of AlphaFold2 in the Critical Assessment of Protein Structure (CASP) prediction exercise,^[^ ^82^ ^]^ many groups have attempted to extend AlphaFold2, or to use it in new and creative ways. OpenFold is essentially a reimplementation of AlphaFold2 using Pytorch ^[^ ^83^ ^]^ instead of Tensorflow.^[^ ^84^ ^]^ Unlike AlphaFold, OpenFold comes with source code for model training,^[^ ^85^ ^]^ and a large set of precomputed alignments with associated structures.^[^ ^86^ ^]^ FastFold also re‐implements AlphaFold2, with an emphasis on faster training.^[^ ^87^ ^]^ RosettaFold differs more from AlphaFold2 than OpenFold and FastFold. It replaces the AlphaFold2 two‐track (MSA and pair representation) Evoformer with a three‐track NN that lets the model reason simultaneously in one, two and three dimensions.^[^ ^88^ ^]^ Unfortunately, this conceptually appealing modification does not seem to help and may even be detrimental, since benchmarks indicate that RosettaFold cannot fully compete with AlphaFold2 on the CASP targets.^[^ ^65^ ^]^

Many groups have also made efforts to exploit the AlphaFold2 breakthrough for tasks other than structure prediction of protein monomers. Uni‐Fold is a variant of AlphaFold2 for symmetric complexes.^[^ ^89^ ^]^ AlphaFold2 Multimer allows the modeling of protein complexes based on the input stoichiometry.^[^ ^56^ ^]^ Further developments extend the use of AlphaFold2 to very large protein complexes that exceed the length limits of the protein language model.^[^ ^90^ ^]^ The AlphaFold2 Multimer interface prediction is reliable enough to be used in computational screens for interaction partners of a protein of interest.^[^ ^57^ ^]^ By restricting the MSA to sub‐alignments containing only sequences that cluster based on pairwise similarities,^[^ ^91^ ^]^ AlphaFold2 can be tuned to model different protein conformations.^[^ ^92^ ^]^ Thus, AlphaFold2 even provides (indirect) insights into protein dynamics.

### Structure prediction from single sequences: ESMFold, OmegaFold, RGN2

There are far more protein sequences ^[^ ^93^ ^]^ than experimental protein structures.^[^ ^81^ ^]^ Is it possible to learn about protein structure, without recourse to experimental structural information? The precedent of the language models suggests that it is. Language models are trained on large amounts of unlabeled text, for relatively simple surrogate tasks, such as next word prediction, or masked token prediction. Yet this training is useful for seemingly more challenging tasks, such as translation, suggesting that some understanding of the “meaning” of language is developed during the training.

ESMFold translates this observation from human language to proteins ^[^ ^94^ ^]^ (Figure 7B). It is based on a protein language model called ESM1 (Evolutionary Scale Modeling) for the smaller version ^[^ ^95^ ^]^ and ESM2 for a larger, later version with about 100 times more parameters than AlphaFold2.^[^ ^95^ ^]^ The ESM and ESM2 language models are trained exclusively on single protein sequences, using a masked language modeling objective. Multiple sequence alignments, or protein structures are not used at any point in LLM training. Nevertheless, the models learn enough about proteins to be able to extract sufficient “meaning” from a given protein sequence to guide structure prediction. The conversion of the “implicit” structural knowledge of the LLM to concrete 3D coordinates happens in two steps. First, a so‐called Folding block derives a pair representation from the LLM feature vectors, and then reasons over the pair representation. Finally, the Structure block computes concrete 3D coordinates. On single sequences, ESMFold outperforms AlphaFold2 by a wide margin. However, it does not quite match the performance of Alphafold2 when AlphaFold2 is allowed to use multiple sequence alignments.^[^ ^94^ ^]^ Compared to AlphaFold2, ESMFold is conceptually and architecturally simpler, and does not require time‐consuming up‐front steps such as database searches or sequence alignments. As a result, predictions are faster than for AlphaFold2, despite the vastly greater model size. Currently, over 700 million structures that have been pre‐calculated by ESMFold are available from the ESM Metagenomic Atlas.^[^ ^94^ ^]^

OmegaFold is conceptually very similar to ESMFold.^[^ ^96^ ^]^ It consists of a language model, a block that reasons over the pair representation called the Geoformer, and a Structure module. With 670 million parameters, OmegaFold is comparable in size to ESM1, and halfway between AlphaFold and ESMFold (on a logarithmic scale). Like ESMFold, OmegaFold is intended for the use with single protein sequences, without recourse to evolutionary information, and outperforms AlphaFold2 in this category.^[^ ^96^ ^]^ RGN2 departs from the three‐module architecture. It features only a relatively small protein language model, termed AminoBERT, and an innovative Structure module that describes protein structure in a very principled way.^[^ ^97^, ^98^ ^]^ Like ESMFold and OmegaFold, RGN2 is intended for single sequences. However, it only performs comparably with AlphaFold2 without evolutionary information,^[^ ^99^ ^]^ and less well than ESMFold or OmegaFold.^[^ ^94^, ^96^ ^]^

ESMFold, OmegaFold and RGN2 share a clean separation between the LLM and the other parts of the NN. At least initially, the LLM is trained independently of structural information. Recently, attempts have been made to inject structural information into LLM training using different types of GNNs.^[^ ^100^, ^101^, ^102^ ^]^ In addition, ideas from diffusion have been adapted for model pre‐training.^[^ ^102^ ^]^ Typically, the structure‐enhanced protein LLMs perform slightly better than pure protein LLMs in tasks such as GO category prediction or EC number prediction.^[^ ^101^, ^102^ ^]^ However, improvements are relatively modest, perhaps because protein LLMs already have structural information implicitly, and therefore do not need it explicitly. Advantages of jointly trained LLMs over vanilla LLMs for structure prediction from sequence data alone have not been demonstrated, and may not be expected.

ESMFold and OmegaFold are better at predicting structure from a single sequence without evolutionary information than AlphaFold2 (used “off‐label”, without access to related sequences). However, even ESMFold and OmegaFold still do not solve the Anfinsen task of folding a protein,^[^ ^103^ ^]^ since the in silico folding trajectory is completely non‐physiological. Currently, the deep learning methods for structure prediction outperform physics based methods. They make the folding process less likely to get stuck in local minima,^[^ ^104^ ^]^ presumably by creating an effective folding landscape that is less rugged than the true physical folding landscape.^[^ ^105^ ^]^

### Structure prediction for proteins, nucleic acids, and small molecules: AlphaFold3

AlphaFold3 improves AlphaFold yet again, mostly by making it more broadly applicable ^[^ ^106^ ^]^ (Figure 7C). AlphaFold2 has been designed for protein structure prediction only. AlphaFold3 can also handle nucleic acids (DNA and RNA) and small molecule ligands. Like AlphaFold2 and ESMFold, AlphaFold3 is divided into an Evoformer like module that essentially reasons in sequence space, and a structure module that converts an enriched sequence representation into a 3D structure. The design changes in the Evoformer module in AlphaFold3 compared to AlphaFold2 are relatively moderate. The MSA is still used, but de‐emphasized, in favor of the pair representation. The major design changes between AlphaFold2 and Alphafold3 are in the Structure module. Here, AlphaFold3 does away with almost everything that was believed to make AlphaFold2 successful.^[^ ^82^ ^]^ The Structure module of AlphaFold3 is basically a DDPM. However, this DDPM does not work with images. Instead, it deals with mappings from a molecular graph (with atoms as vertices and bonds as edges) to the space of 3D coordinates. Since these mappings are technically known as Molecular Conformer Fields (MCFs), the Structure module can be described as an Evoformer guided MCF denoising network.^[^ ^107^ ^]^ As every DDPM, the Structure module of AlphaFold3 requires a NN to control the denoising. PerceiverIO, a domain agnostic network with a transformer architecture in latent space was chosen (instead of the usual U‐Net, which would not be suitable to operate on fields).^[^ ^108^ ^]^ A Perceiver based network is computationally efficient due to latent space compression,^[^ ^109^ ^]^ and well suited to the goal of modeling a diverse set of molecules (proteins, DNA, RNA, and small molecules). Currently, AlphaFold3 is available only through a server.^[^ ^110^ ^]^ However, DeepMind has promised to release the source code and model weights to the research community.

### Structure prediction tools know when they get it right and when they do not

Some NN‐based structure prediction tools have the ability to provide realistic estimates of the accuracy of their own predictions. Although there is no “ground truth” reference structure, the quality measures are based on quality indicators that were originally developed for comparisons between test and reference structures. The Root Mean Square Deviation (RMSD) and Template Modeling scores measure overall accuracy, based on an optimal superposition. The RMSD gives a lot of weight to the worst discrepancies, which can mask otherwise good agreement. The TM score avoids this problem by weighting discrepancies based on the Gerstein‐Levitt score.^[^ ^111^ ^]^ TM scores range from 0% (worst agreement) to 100% (best agreement). A variant of the TM, the interface Template Modeling score (iTM), is a global measure of interface accuracy. Like the TM scores, iTM scores range from 0% (worst match) to 100% (best match). The Local Distance Difference Test (LDDT) measures local structural quality, on a per‐ residue basis, without superposition. The LDDT is defined as the proportion of correct local distances and takes values between 0% (all distances are incorrect) and 100% (all distances are correct).^[^ ^112^ ^]^ Finally, the Frame Aligned Point Error (FAPE) measures the (clipped) error of pairwise distances and is very useful in the context of protein design.^[^ ^113^ ^]^ In the context of structure prediction, the scores are estimated in the absence of a reference structure and are therefore only predictions. This is indicated by the extra “p” in the names of the pTM, ipTM, pLDDT, and pFAPE scores.^[^ ^55^, ^56^ ^]^

### Assistance with experimental structure determination

For at least half a century, experimental macromolecular structures, determined by X‐ray crystallography, cryo‐EM, and NMR have served as a gold standard reference for the development of protein structure prediction tools. Ironically, this development is now coming full circle, with AI tools being developed to help with experimental structure determination. In X‐ray crystallography, AlphaFold2 models are of sufficient quality to be used in molecular replacement, to solve the phase problem.^[^ ^114^ ^]^ ModelAngelo automatically interprets cryo‐EM maps.^[^ ^115^ ^]^ It uses a CNN to predict protein and nucleic acid residues, a GNN to optimize positions and orientations, and finally a post‐processing step to complete a model of a quality that rivals the quality of models built by human experts.^[^ ^115^ ^]^

Prior expectation has always affected experimental structure determination to some degree. In X‐ray crystallography, molecular replacement models have been used to solve the phase problem.^[^ ^116^ ^]^ Moreover, prior chemical information, in the form of target values for bond lengths and angles, has been universally applied in refinement.^[^ ^117^ ^]^ Cryo‐EM experimental maps are not always “model‐free” either, particularly when models have been used to aid particle picking prior to classification and 3D reconstruction.^[^ ^118^ ^]^ While the use of prior knowledge is in principle a good thing, its excessive use also creates a problem. In X‐ray crystallography (and also cryo‐EM), Ramachandran angles are typically deliberately not refined, in order to be able to use them later on as a criterion for the correctness of a structure at the validation stage.^[^ ^119^ ^]^ With the availability of increasingly sophisticated modeling algorithms, the community will have to think about new ways to judge to what extent prior expectations bias “experimental” structures. Perhaps ideas from the field of DeepFake detection ^[^ ^120^ ^]^ could help to judge to what extent a published structure is “experimental,” and to what extent its features are overly based on expectation.

### Inverse protein folding

Inverse folding is the reverse of the structure prediction task.^[^ ^121^ ^]^ Given a structure, the goal is to find sequences that are compatible with a given protein backbone structure. Conceptually, this is similar to translation, from the “language” of protein backbone features to the “language” of protein sequence. From this perspective, a transformer‐like architecture with encoder and decoder (Figure 3G) is natural.

**FIGURE 8:**
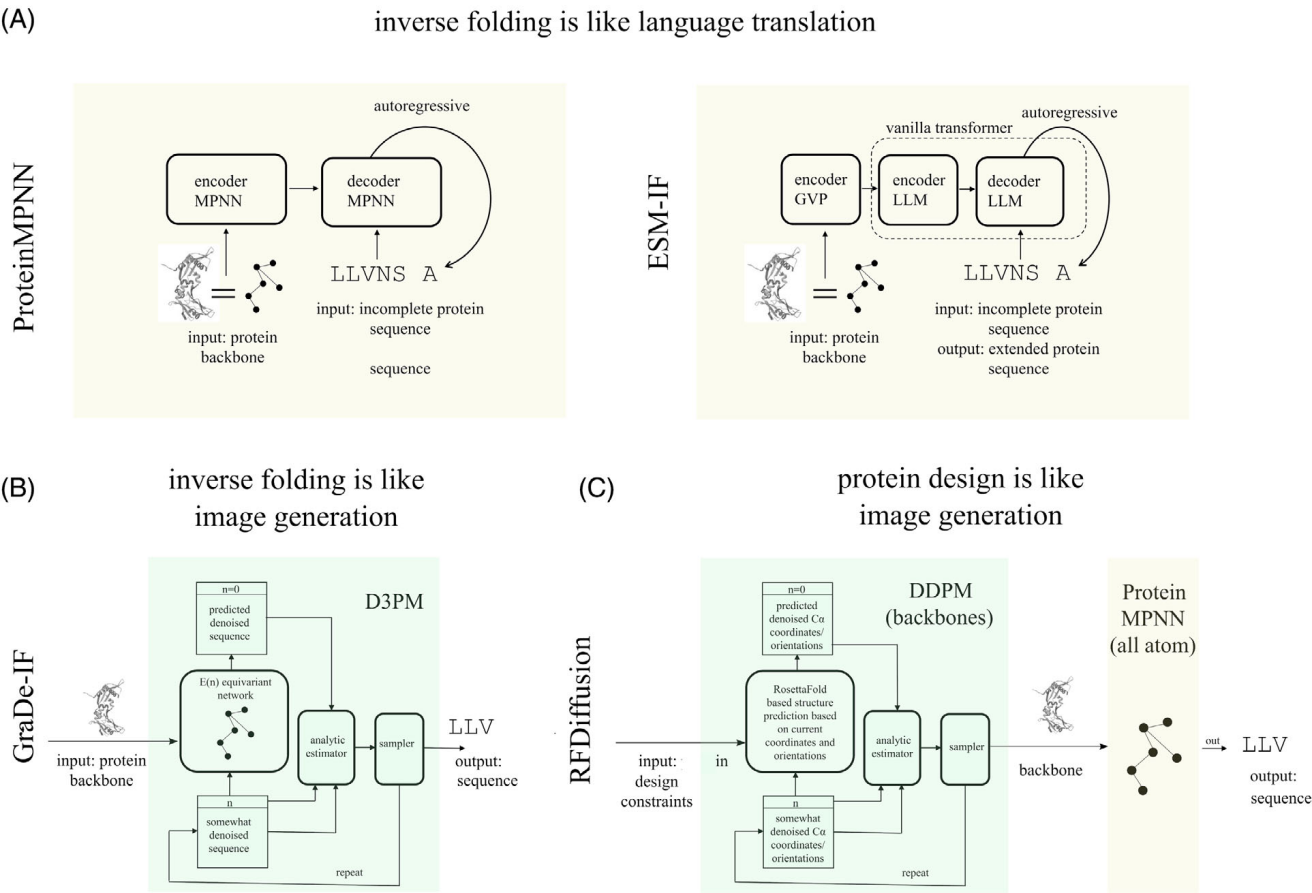
**Inverse folding and protein design tools**: (A) The inverse folding problem can be treated like a language translation task, with encoders and decoders. ProteinMPNN uses GNN encoders and decoders, ESM‐IF uses a GNN only for the encoding step and a vanilla transformer for the decoding step. (B) The inverse folding problem can also be treated like an image generation problem. GraDe‐IF uses a D3PM to denoise a protein sequence, conditioned on a protein backbone structure as the input. (C) The protein design problem can be broken down into a protein backbone design problem and an inverse folding problem. The backbone design problem can be treated like an image generation problem. RFDiffusion uses a DDPM that operates on residue frame positions and orientations. LLM modules and their graph analogs and on yellow background, denoising diffusion based modules are on green background.

For the encoding stage, proteins are naturally represented as graphs, with the amino acids as nodes (also called vertices), and edges that are informative about proximity (typically, any node is connected by edges to the 30–40 nodes representing the spatially closest neighbors). Both nodes and edges can be associated with feature arrays, also termed hidden states. Those of nodes encode information about the local environment of a node. Those of edges encode pairwise information, such as pairwise distances between backbone atoms, or relative orientations between residue‐associated coordinate frames. In the absence of edge features, a GCN (Figure 5B), GAT (Figure 5C) or GVP (Figure 5E) can be used as the encoder. If edge features are present, an MPNN (Figure 5D) or E(n) equivariant network (not shown) needs to be used (Figure 8A).

For the decoding stage, there is considerably more variation than for the encoding stage. ESF‐IF simply conditions a vanilla ^[^ ^6^ ^]^ LLM decoder on the encoder hidden states.^[^ ^122^ ^]^ By contrast, ProteinMPNN ^[^ ^123^ ^]^ and its predecessor ^[^ ^124^ ^]^ stay with the graph architecture also for the decoder. For both choices, decoding is autoregressive, either from one end of the protein to the other, or in random order to avoid systematic design constraints from a directional design process (Figure 8A). Such auto‐regressive modeling is very successful in the domain of human language. However, it is not optimal for proteins, in part because it is inefficient. AlphaDesign ^[^ ^125^ ^]^ addresses this problem by introducing confidence scores for each residue. Amino acids can then be assigned concurrently, instead of auto‐regressively. Several iterations are required. At every step, highly constrained amino acids from the previous round strongly influence the choice of other amino acids, whereas the more arbitrary choices from the previous round have less influence. In practice, switching from auto‐regressive to simultaneous sequence assignment speeds up the design process by almost two orders of magnitude.^[^ ^126^ ^]^ The gradual refinement of a noisy protein sequence by AlphaDesign is reminiscent of (but technically distinct from) diffusion denoising, blurring the line between encoder/decoder and denoising diffusion based approaches. PiFold does away altogether with multiple iterations and predicts protein sequence in a one‐shot way.^[^ ^126^ ^]^

Instead of considering the inverse folding problem as a translation task, it can also be viewed as a task in generative modeling, similar to image generation, with the protein backbone as the “language prompt” and the amino acid sequence as the “image.” GraDe‐IF ^[^ ^127^ ^]^ takes this approach. It uses a D3PM to denoise protein sequences, conditioned on the output of an E(n) equivariant graph neural network ^[^ ^54^ ^]^ (Figure 8B). Training is based on proteins of known structure that have their amino acid sequences stochastically transitioned to other sequences, using BLOSUM matrices ^[^ ^128^ ^]^ with varying temperatures as the transition kernel.

As LLMs and D3PMs learn the probabilities of protein sequences given a fold, they can also learn relative probabilities of sequences for noised and actual protein backbones. Indirectly, they thus learn folding potentials, according to Boltzmann's rule.^[^ ^129^ ^]^ In principle, these quasi‐physical potentials could be used to improve sequence predictions.^[^ ^130^, ^131^ ^]^ In practice, however, improvements are modest. For both protein LLMs and D3PMs, sequence recovery rates are ∼30%–50%, depending on how sequence recovery is scored.^[^ ^127^ ^]^

Besides the LLM and D3PM approaches to the inverse folding problem, other approaches are possible as well. AF2‐design (not to be confused with AlphaDesign) inverts the AlphaFold2 structure prediction network, and chooses the protein sequence to maximize confidence scores for a given backbone structure.^[^ ^132^ ^]^ The task of fitting amino acid side chains into a fold can be compared to a Sudoku puzzle. An inverse folding tool combines this idea with the use of GNNs.^[^ ^133^ ^]^ The inverse folding problem can also be approached from a strictly Bayesian point of view.^[^ ^134^ ^]^ Finally, the problem can be treated as a shape‐matching problem. The local sequence is deduced by finding suitable templates in a database of protein backbones with known sequences.^[^ ^135^ ^]^ According to the authors, the sequence recovery for this approach is higher than for template‐independent methods.

For inverse folding to be broadly applicable, there should be an option to keep sequences of multi‐copy subunits in homo‐oligomers, or more generally protein complexes, consistent. Moreover, the user should have the possibility to keep parts of the amino acid sequence constant. Designs with a partly fixed sequence may be needed to preserve a ligand binding site or enzyme active site, or to optimize the binding of a designed protein to a fixed target. ProteinMPNN offers options for multimer design and for fixed amino acids out of the box.^[^ ^123^ ^]^ For other inverse folding tools, some tinkering with the code may be necessary. Perhaps the most relevant biotech application of inverse folding is antibody sequence design.^[^ ^136^ ^]^ With AntiFold, there is now even a specialized inverse folding tool for this purpose.^[^ ^137^ ^]^ However, any design successes must be interpreted with caution. LLM guided antibody maturation, combined with minimal evolution in the laboratory, can be sufficient to improve antibody binding affinity.^[^ ^138^ ^]^ In the antibody maturation study, inverse folding was not used. Instead, success was achieved without explicit knowledge of the antibody or the target, strongly suggesting that “many roads lead to Rome” when it comes to antibody improvement.^[^ ^138^ ^]^

### Protein design

In protein design, both the protein sequence and the protein structure are varied. The simplest form of design is to “hallucinate” new proteins, based on some notion of the probability distribution for “real” proteins. Constraint‐free design is easy with LLMs in autoregressive mode and has been done using either ESMDesign, or ProtGPT2.^[^ ^139^, ^140^ ^]^ The resulting protein sequences are only distantly related to natural ones. Most of them are predicted to encode folded, globular proteins. Unconstrained protein design has also been carried out using Generative Adversarial Networks (GANs),^[^ ^141^ ^]^ the state of the art technology for image generation prior to the advent of DDPMs.^[^ ^142^ ^]^ DDPMs are also very suitable for design, because they ultimately learn the distribution of naturally occurring proteins and their structures. DDPMs can be used in torsional space. Equivariance is then automatic.^[^ ^143^ ^]^ Alternatively, DDPMs can also be used in Cartesian space, together with rotation/translation invariant decoders.^[^ ^144^ ^]^ For proteins, a decoder similar to the Invariant Point Attention (IPA) decoder of the Alphafold2 Structure module has been used.^[^ ^145^ ^]^

To be practically useful, protein design needs to be steerable into desirable directions. For example, part of a protein structure may be provided as a fixed input, with the goal to design the rest. This task is similar to image in‐painting, a subfield of image generation that requires a NN to complete a partially masked image.^[^ ^146^ ^]^ Translated to proteins, sequence and structure in‐painting require a structure prediction network that is trained to predict not only structure, but also masked regions of protein sequence.^[^ ^147^ ^]^ Currently, the most versatile tool for protein design is RFDiffusion ^[^ ^113^ ^]^ (Figure 8C). RFDiffusion applies the idea of Gaussian noising to Cα‐coordinates and also generalizes it to residue frame rotations.^[^ ^148^, ^149^ ^]^ RFDiffusion builds on a variant of RosettaFold ^[^ ^88^ ^]^ to guide the denoising process, and computes only protein backbones. For sequence design, RFDiffusion depends on inverse folding tools, such as ProteinMPNN.^[^ ^123^ ^]^ RFDiffusion is suitable for a broad spectrum of design tasks. Partial diffusion can generate sequence and structure variety around a core fold. Scaffolding designs a protein backbone for a fixed motif, for example an enzyme active site or a ligand binding site.^[^ ^150^ ^]^ DDPMs also excel at designing polypeptides with a high affinity to a given protein.^[^ ^151^ ^]^

### Small molecule ligand design

The task of estimating the affinity of a small molecule ligand to a protein the is known in the machine learning community as the protein compound interaction (CPI) problem. Traditionally, this task required the availability of an accurate protein structure. This structure could then be used to dock the ligand to the protein, using tools such as AutoDock.^[^ ^152^ ^]^ For each docking pose, the affinity of the ligand to the protein could then be estimated, either using the scoring functions of the docking tools, or using separate predictors such as xscore.^[^ ^153^ ^]^ With the advent of machine learning, it is now possible to estimate the affinity of a ligand to a protein given only the protein sequence and a 1D‐representation of the ligand, such as a SMILES string.^[^ ^154^ ^]^ Typically, such systems use CNNs to reason over the amino acid sequence,^[^ ^155^, ^156^, ^157^ ^]^ and CNNs, or various types of GNNs, to reason over 1D‐representation of the small molecule ^[^ ^155^, ^156^ ^]^ or its graph equivalent.^[^ ^157^ ^]^ The output of the CNNs (or the CNN and GNN) can be processed by a simple densely connected NN, or by more sophisticated means, for example using an attention mechanism.^[^ ^158^ ^]^ TransformerCPI treats the affinity prediction problem by analogy to language translation, with a transformer encoder‐decoder architecture, but without auto‐regression.^[^ ^159^ ^]^ PerceiverCPI also ultimately builds on the attention mechanism.^[^ ^160^ ^]^ However, it is modelled on the Perceiver architecture ^[^ ^108^, ^109^ ^]^ that has also inspired the architecture of the Structure module of Alphafold3.^[^ ^106^ ^]^

Given a target structure, ligands can not only be evaluated for affinity, but also designed from scratch. Currently, the predominant approach relies on denoising diffusion, using DDPMs to denoise the coordinates of small molecule atoms, and D3PMs to denoise atom types. The protein structure is used to condition the denoising process, similar to the way a text prompt conditions image generation by a DDPM. DiffBP ^[^ ^161^ ^]^ and DecompDiff ^[^ ^162^ ^]^ implement these ideas. DiffBP designs the entire small molecule in one go.^[^ ^161^ ^]^ DecompDiff is inspired by fragment based drug design.^[^ ^163^ ^]^ It uses DDPMs/D3PMs to design fragments that bind to individual pockets of the protein, and then proceeds to connect these fragments.^[^ ^162^ ^]^ The Pocket based Molecular Diffusion Model (PMDM) ^[^ ^164^ ^]^ is conceptually similar to DiffBP. It uses cross‐attention for the conditioning process, in a manner that is reminiscent of how SD uses cross‐attention to condition image generation by a text prompt.^[^ ^36^ ^]^

### Democratizing the power of deep learning

**FIGURE 9:**
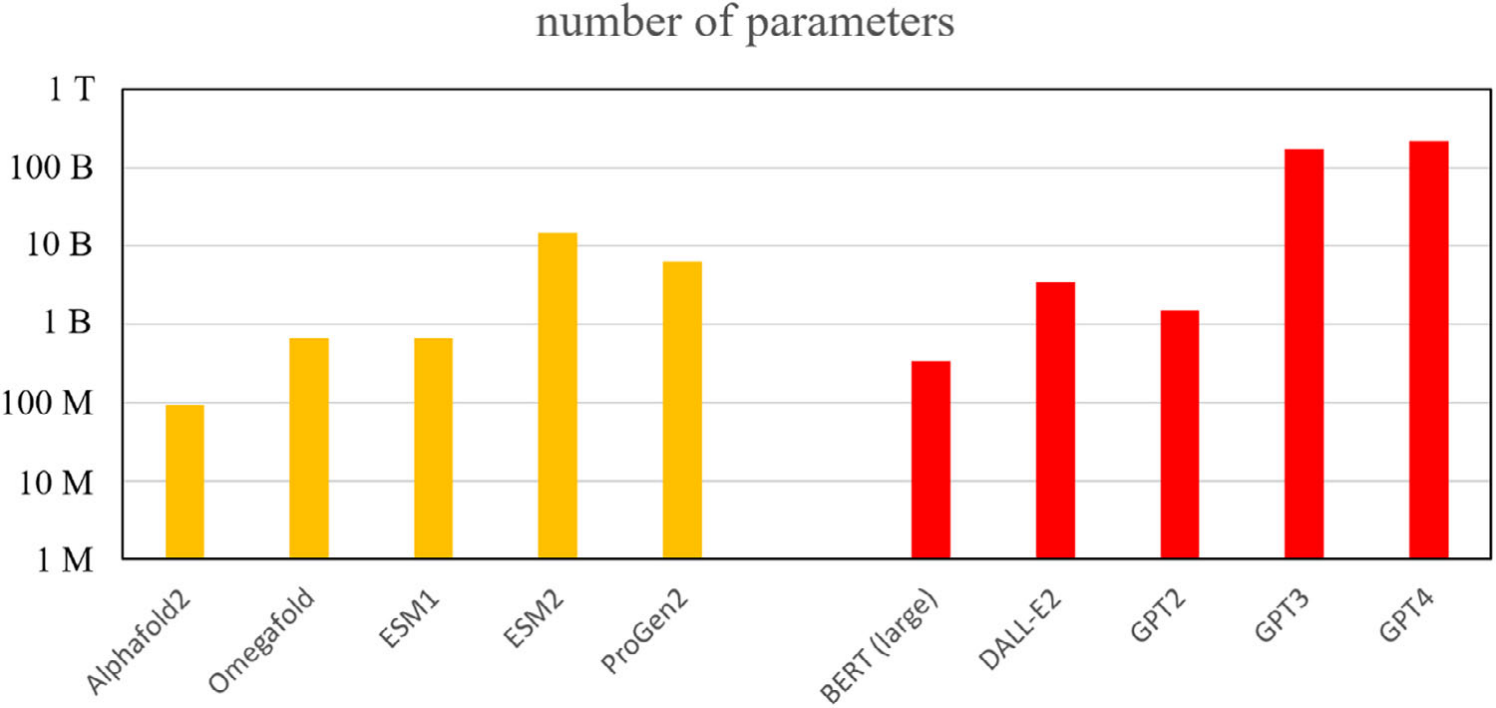
**Number of parameters of AI models for protein structure prediction**: Protein specific models are in orange, general purpose models are in red. “M” stands for million (10^6^), “B” for billion (10^9^), and “T” for trillion (10^12^). Note the logarithmic scale on the y‐axis. The largest general purpose models (GPT3, GPT4) are larger than the largest dedicated protein models, but the difference is only roughly an order of magnitude.

Phil Anderson's classic physics motto “More is different” ^[^ ^165^ ^]^ has a counterpart in deep learning: “More is better”.^[^ ^166^ ^]^ Significant performance benefits can be gained from larger models and more training.^[^ ^167^, ^168^ ^]^ The empirical scaling rules have fueled a gold rush for models with more and more parameters, and for larger and larger training datasets (Figure 9). This trend has a dark side. Apart from the ecologic cost,^[^ ^169^, ^170^ ^]^ it creates monopolies or oligopolies for large companies that have sufficient computing power (GPUs and TPUs) and financial resources to pay the massive electricity costs for training large models.^[^ ^171^ ^]^ Fortunately, model‐based inference for a single sequence is orders of magnitude cheaper than model training. All that is required is enough random access memory (RAM/VRAM) to hold the large models in memory, or software to work with models that can only be partially loaded.^[^ ^172^ ^]^ In practice, it is now possible to run massive LLMs, such as Facebook's ESM2, on a single compute server. This has inspired community efforts to make the large models widely available so that developers can build on existing models. HuggingFace has streamlined this process making it easy to obtain and run the models, even in an academic environment with limited computing resources.^[^ ^173^ ^]^ In addition, machine learning researchers can now often avoid the tedious task of dealing with software dependencies on local hardware by using docker containers,^[^ ^174^ ^]^ or notebooks in the cloud such as Google Colab.^[^ ^175^ ^]^

## CONCLUSIONS

Protein structural biology is in a period of profound change. Many researchers of my own generation, and also some of the professional societies, have reacted to the changes with a mixture of denial, concern, and even apprehension. For the author, it was an eye‐opening experience to see the reactions of the founders of modern structural biology. Unlike the author's generation, who had entered an increasingly “established” and “secure” field, this older generation –in their own youth– had ventured into the unknown, presumably fully aware that failure was the most likely outcome. Unlike everyone else, this group enthusiastically welcomed the change. One of them showed, almost demonstratively, Max‐Perutz's collection of letters “What a time I am having”,^[^ ^176^ ^]^ about the changes in structural biology that Perutz shaped and witnessed many decades ago. As the experimental structural biology community, let's learn from the attitudes of the founders. Let's embrace the new developments, even if they make some of our hard‐earned and cherished skills obsolete. Let's see the sea‐change in our field as an opportunity! And let's adapt our curricula to train a new generation of structural biologists to take full advantage of the revolution that is happening before our eyes!

## CONFLICT OF INTEREST STATEMENT

The author declares no conflicts of interest.

## Data Availability

New data were not generated for this review.
